# Development and testing of a public health emergency intelligence analysis system based on text analysis and NLP analysis

**DOI:** 10.3389/fpubh.2025.1677306

**Published:** 2025-10-16

**Authors:** Feng Yang, Xingxi Huang, Wencheng Huang, Tao Jiang

**Affiliations:** School of Humanities and Management, Guilin Medical University, Guilin, China

**Keywords:** public health emergencies, knowledge graph, health education resources, technology acceptance model, epidemic notification, library and information science

## Abstract

**Objective:**

To tackle challenges including delayed information support and inefficient decision-making in public health emergency response, this study develops an intelligence analysis system for public health emergencies based on emergency information management theory from library and information science.

**Methods:**

Using 1,026 text data items such as government reports and flow survey records from the COVID-19 epidemic in Shijiazhuang City (1,033 confirmed cases), multimodal analysis methods were integrated, including logistic regression, C5.0 decision tree, TransH-based knowledge graph, and chi-square test. The BIO tagging scheme was adopted with annotations performed by three epidemiology professionals, achieving an inter-annotator agreement (Kappa) of 0.78.

**Results:**

Key transmission sites were identified by chi-square test (*χ*^2^ = 87.32, *p* < 0.001). Risk factors such as advanced age (OR = 3.15) and village clinic visits (OR = 4.72) were identified through logistic regression. A case-place-time network was constructed using the TransH algorithm (accuracy 0.89). The C5.0 decision tree classified high-risk areas (AUC = 0.91), and Apriori association rules revealed patterns such as “wedding banquet → family gathering” (confidence 0.86). A Python-based system improved intelligence extraction efficiency by 47.8%.

**Conclusion:**

The study successfully establishes an interdisciplinary framework integrating library informatics, epidemiology, and AI. It identifies churches and wedding banquets as key transmission nodes, and village clinics as amplifiers due to delays in identification and reporting. The developed software tool enhances response efficiency, supporting rapid contact tracing and control strategy formulation.

## 1 Background

Public health emergencies pose severe challenges to the timeliness of intelligence response due to their unpredictability and high harm. The COVID-19 epidemic in Shijiazhuang at the beginning of 2021 showed that hidden transmission of the epidemic in rural areas was a significant public health risk: before the epidemic was discovered, 33.02% of the cases did not seek medical treatment or only went to the village clinic, resulting in the virus forming a cluster transmission chain at home (74.27%), school (15.64%) and religious places (average 82.67 cases/place). Traditional epidemic analysis relies on manual epidemiological survey reports; when confronted with a daily average of thousands of cases, it suffers from delays in intelligence dissemination and fragmented information.

The theoretical methods of library and information science in information ordering, knowledge organization and intelligence services can provide new ideas for responding to public health emergencies. During the COVID-19 pandemic, the National Library of Medicine (NLM) integrated multi-source data through the Disaster Information Management Research Center (DIMRC) to build a thematic knowledge base, increasing the average daily service volume by 300%. This study integrates the core theory of library and information science with natural language processing technology to develop an intelligent epidemic analysis system, which is of great practical significance for improving our country’s public health emergency intelligence response capabilities.

## 2 Research progress at home and abroad

The efficient response to public health emergencies relies on the rapid processing and accurate analysis of massive heterogeneous information. Traditional public health monitoring systems have significant limitations in real-time, coverage and information depth, making it difficult to meet the needs of complex and changeable emergency decision-making. In recent years, the rapid development of natural language processing (NLP) and text analysis technology has provided a new technical path for automatically extracting epidemic intelligence from multi-source text data (such as social media, electronic medical records, and news reports). These technologies not only enable early warning, but also support critical decision-making links such as public opinion guidance, resource allocation, and risk communication. This paper systematically sorts out the research progress in this field at home and abroad, focusing on the technological breakthroughs and application practices in core directions such as intelligence extraction, public opinion evolution analysis, drug safety monitoring and early warning decision support, so as to provide a theoretical reference for future system optimisation.

### 2.1 Domestic research progress

#### 2.1.1 Intelligent intelligence extraction and semantic understanding

Domestic research teams have made significant breakthroughs in the field of intelligence extraction of multi-source heterogeneous texts. In 2025, the “Public Health Research Text Analysis System” developed by Ni Pengfei’s team will achieve high-precision semantic preservation of survey texts through a three-stage processing process of style shielding, core extraction, and style reconstruction (1). The system first identifies and blocks the stylized expressions in the original text, extracts the core information paragraphs, and then reconstructs them according to the target style requirements, which solves the semantic distortion problem of traditional methods in style transfer and significantly improves the accuracy of intelligence analysis (2). In terms of clinical text analysis, the Korean emergency triage study showed that the KLUE-RoBERTa-based model reached an F1 value of 0.965 in the symptom recognition task, and the decision-making process was visualized through explainable artificial intelligence (XAI) technology (such as the SHAP method), so that the model output was highly trusted by clinicians, and the Jaccard similarity reached 0.722 (3).

Knowledge-driven intelligence enhancement is another important direction. Yang Guoping’s team from the Third Xiangya Hospital of Central South University has built an intelligent drug adverse reaction monitoring system (AIMES), which innovatively integrates deep learning and knowledge graph technology (4). The system uses knowledge graph inference to complete missing drug descriptions in electronic medical records and automatically screen potential adverse reaction signals (5). After actual deployment and verification, the adaptability of the system to Chinese electronic medical records is significantly better than that of international common tools, and the related technology has been authorized by the national patent (ZL202210102746.8).

#### 2.1.2 Analysis of public opinion evolution and theme mining

Social media public opinion analysis has become a key area of domestic research. In 2023, Wang Tong’s team from Lanzhou University of Finance and Economics proposed the SnowNLP-LDA fusion model, which divides the public opinion cycle into three stages of “formation-outbreak-decline” through sentiment analysis, and identifies the core issues of each stage in combination with the LDA theme model (6). Through the analysis of 87,692 Weibo comments, the study revealed the polarization of public sentiment after the policy was released, providing a quantitative basis for the government to accurately guide public opinion (7). This method was verified in the analysis of public opinion on the “New Ten Articles” epidemic prevention and control policies in 2023, and found that the public’s attitude toward the policy showed the characteristics of “high recognition of supportive clauses” and “highly controversial operational clauses” (8).

The team led by Zeng Ziming at Wuhan University has further optimized the public opinion analysis framework and proposed a BERT-BiLSTM-Attention composite model (9). This model leverages BERT’s deep semantic representation capabilities, BiLSTM’s advantages in temporal modeling, and the attention mechanism’s focus on key information, achieving dynamic tracking of public opinion themes and sentiment evolution. Research indicates a strong correlation between offline viral mutation events and online sentiment fluctuations, providing theoretical support for ‘online-offline linked early warning’ (10) (Table 1).

**Table 1:** Representative research projects and technical characteristics in China.

Research team	System/model name	Core technology	Application scenarios	Data validation
Ni Pengfei Team (2025)	Public health research text analysis system	Style shielding and reconstruction mechanisms	Analysis of government research reports	Increased Style Swap Accuracy by 35%
Yang Guoping Team (2023)	AIMES system	Deep learning + knowledge graph	Adverse drug reaction monitoring	Chinese electronic medical record recognition F1 value 0.92
Wang Tong Team (2023)	SnowNLP-LDA model	Sentiment analysis + thematic modeling	Weibo public opinion evolves	87,692 comments were divided into periods
Zeng Ziming Team (2023)	BERT-BiLSTM-Attention	Composite neural networks	The theme of public opinion evolves	Correlation analysis of virus mutation events

#### 2.1.3 Drug safety and adverse reaction monitoring

Drug safety monitoring is a critical part of public health intelligence analysis. The AIMES system (AdversedrugreactionIntelligentMonitoringandEvaluation System) developed by Xiangya Third Hospital solves three major problems in adverse reaction signal extraction in Chinese electronic medical records through the combination of knowledge graph embedding and deep learning: terminology diversity, descriptive unstructured and semantic ambiguity (11). A large-scale pharmaceutical knowledge graph containing 500,000 nodes was systematically constructed, covering the entity relationship between drugs, indications, and adverse reactions, which significantly improved the sensitivity of identification of rare adverse reactions (12).

In terms of vaccine safety monitoring, a new method developed by the University of Copenhagen in collaboration with Chinese researchers uses natural language processing to automatically extract signs and symptom descriptions from VAERS (Vaccine Adverse Event Reporting System) and map them to the MedDRA terminology system with the help of GPT-3.5. This method improves signal processing efficiency by 40% and reduces manual review workload by 17%, providing a feasible solution for mass vaccination safety monitoring (13).

#### 2.1.4 Early warning decision support system

In terms of vaccine safety monitoring, the University of Copenhagen has developed new methods in collaboration with Chinese researchers in the field of early warning and decision support, and reinforcement learning and modeling of complex systems have become emerging research directions. The “AI Q-learning Public Health Early Warning Framework” proposed in 2024 simulates the dynamic game of epidemic transmission and intervention through state-action reward mapping, achieving accurate identification of high-risk areas (14). The model encodes regional population flow, medical resource density and real-time infection data into state space, and optimizes intervention strategies such as isolation intensity and material allocation through Q value iteration, reducing the resource misallocation rate by 28% in the simulated environment. It uses natural language processing to automatically extract signs and symptom descriptions from VAERS (Vaccine Adverse Event Reporting System) and maps them to the MedDRA terminology system with the assistance of GPT-3.5 (15). This method improves signal processing efficiency by 40% and reduces manual review workload by 17%, providing a feasible solution for mass vaccination safety monitoring.

### 2.2 International research progress

#### 2.2.1 Multi-source data fusion and real-time analysis

International research focuses on the collaborative analysis of multi-source heterogeneous data. The AI-Driven Epidemic Intelligence framework proposed by the Canadian research team integrates large language models (LLMs), multilingual NLP, and optimisation algorithms to achieve cross-source correlation analysis of news, academic literature, and social media text (16). The system automatically generates a spatio-temporal map of the epidemic transmission chain through entity recognition and event extraction technology, providing support for early warning (17). The 2025 verification shows that the system advances the early warning time of the Ebola epidemic by 14 days from the official WHO notification.

The value of Wikipedia as an open source intelligence source is further explored. The researchers used named entity recognition (NER) technology to automatically extract key indicators such as the number of cases, deaths, and hospitalisations from Wikipedia’s disease entries, and constructed a time series that is highly consistent with official data (18). The 2014 Ebola outbreak in West Africa confirmed that the correlation coefficient between Wikipedia-based surveillance data and real data was 0.93 (*p* < 0.01), providing a low-cost surveillance solution for low-resource areas.

#### 2.2.2 Epidemic prediction and resource optimisation

Social media biosurveillance is widely applied in international research. The Virtual Campus system developed by the Pan American Health Organization (PAHO) enables real-time identification of epidemic outbreak points by analyzing Twitter data from multiple countries (19). This system combines topic clustering and sentiment analysis, allowing it to identify areas of case aggregation as well as assess the public’s mental state, providing a basis for risk communication strategies (20). During the COVID-19 pandemic, this system reached 480,000 users, with an early warning accuracy rate of 82%.

In terms of resource optimisation, text-based prediction models are deeply integrated with operations research methods. In 2025, FrontiersinAI proposed that the epidemic transmission characteristics extracted by NLP can be input into a stochastic optimisation model to dynamically generate medical resource allocation plans. The model comprehensively considers the demand for beds, the risk of infection of medical staff and the stability of the supply chain, and reduces the delay in the admission of critically ill patients by 45% in the simulation application in Lombardy, Italy.

#### 2.2.3 Drug safety and cross-language analysis

Important breakthroughs have been made in cross-language drug safety monitoring. Based on the Dis2Vec model, the disease classification method construction technology solves the problem of term differences in the international adverse drug reaction database through word vector spatial mapping. The application of this model in the EU ADR surveillance network shows that its disease classification accuracy is improved by 19% compared to traditional ICD coding, especially improving the coverage of rare and endemic disease terms.

In the field of clinical text analysis, a study published in the American Journal of Emergency Medicine in 2024 verifies the value of Transformer models in emergency triage. By fine-tuning the clinical BERT model, the system can automatically identify 12 types of symptoms and 2 types of medical history from the doctor-patient dialog text, with an AUROC of 0.893, which significantly reduces the burden of emergency department paperwork.

#### 2.2.4 Social media and multilingual public opinion management

Social media has become a core source of international epidemic intelligence. Research from the perspective of computational linguistics has confirmed that cross-linguistic topic clustering and emotional evolution analysis can accurately identify information dissemination patterns in different cultural backgrounds. A comparative study of Chinese and English social media during the COVID-19 period showed that English users were more concerned about vaccine safety (32.1%), while Chinese users were more concerned about the effectiveness of prevention and control policies (41.7%). This difference provides a scientific basis for localized risk communication.

For disinformation governance, large models such as GPT-3.5 are used to automatically refute rumors and generate text. Through position detection and scientific evidence matching, the system can generate clarification content for false information in real time, and increase the speed of rumor spread decay by 50% in the EU FactCheck platform test (Table 2).

**Table 2:** Internationally representative research projects and technical characteristics.

Country/Institution	System/model name	Core technology	Innovation	Application effect
Canadian Research Team (2025)	AI-Driven Epidemic Intelligence	LLMs + Multilingual NLP	Cross-source data correlation	Ebola warning is 14 days in advance
Pan American Health Organization (PAHO)	VirtualCampus	Topic clustering + sentiment analysis	Social media biomonitoring	Early warning accuracy 82%
EU Research Team	Dis2Vec model	Word vector mapping	Harmonization of terminology across languages	Disease classification accuracy increased by 19%
American Emergency Research (2024)	Clinical BERT fine-tuning model	Transformer Architecture	Emergency symptom recognition	AUROC0.893

### 2.3 Technical challenges and development trends

#### 2.3.1 Existing technical bottlenecks

While text analytics and NLP technologies have made significant advances in public health intelligence analysis, they still face multiple technical challenges:

Data heterogeneity and quality defects: The unstructured nature of social media text, the privacy restrictions of electronic medical records, and the format differences of multi-source data make it difficult to integrate information (19). The analysis of Chinese electronic medical records shows that dialect expressions and abbreviated terms increase the error rate of entity recognition by 15–20%.

Insufficient generalization capabilities: Most models perform well on specific events (e.g., COVID-19) but are less sensitive to emerging infectious diseases (e.g., monkeypox virus) due to lack of training data. The results showed that the average F1 value of the model decayed by 28% after cross-disease migration.

Real-time and accuracy trade-offs: Public opinion analysis systems need to strike a balance between speed and accuracy. The SnowNLP-LDA model has a subject recognition delay of only 10 min in real-time monitoring mode, but at the cost of a 0.12 reduction in topic consistency score (21).

Insufficient utilization of multimodal information: Existing systems focus on plain text analysis, while image reports (such as CT films), audio data (emergency calls), and spatiotemporal trajectories in public health events have not been effectively integrated.

#### 2.3.2 Frontier exploration direction

In the face of the above challenges, researchers are exploring breakthrough paths from multiple dimensions:

Multimodal fusion analysis: Emerging research attempts to integrate text, image, and gene sequence data. For example, the chest X-ray report text is associated with the image features, and a new crown severe prediction model is constructed, so that the prediction window is advanced to 48 h before the onset of symptoms (22).
Small-shot learning and transfer learning: Using pre-training-fine-tuning paradigms, general-domain language models are adapted to public health scenarios with limited annotated data. Yang Guoping’s team adopted a knowledge graph-guided fine-tuning strategy in the AIMES system to increase the F1 value of adverse reaction recognition in small sample scenarios to 0.79.
Causal reasoning and interpretability enhancement: Through causal graph models and counterfactual analysis, the causal chain of risk factors and health outcomes is revealed. The University of Copenhagen study used text data to augment traditional matching analysis, improving bias in treatment effect estimation.
Edge computing deployment: Develop lightweight models (such as pruning BERT) to support localized analysis of grassroots institutions. The African pilot project uses a mobile-optimized NLP model to implement offline symptom report analysis in a low-bandwidth environment (Table 3).

**Table 3:** Comparison of technical challenges and coping strategies.

Technical challenges	Representative solutions	Core innovation	Expected benefits
Data heterogeneity	Knowledge graph embedding	Terminology standardization and relational reasoning	Improve cross-source data fusion capabilities
The algorithm is not generalized	Pre-training - fine-tuning the paradigm	Domain-adaptive transfer learning	The efficiency of the analysis of emerging infectious diseases has been improved
Real-time requirements	Streaming architecture	Incremental LDA vs. online clustering	The delay in public opinion response < 5 min
Multimodal utilization is insufficient	Graph neural network fusion	Text-image joint embedding	Multi-dimensional intelligence correlation analysis

### 2.4 Conclusion and future outlook

Text analysis and NLP technology have become the core driving force of information analysis of public health emergencies, and domestic and foreign research has made significant breakthroughs in the fields of intelligence extraction, public opinion evolution, drug monitoring, and early warning decision-making. However, to achieve the full implementation of the system, it is still necessary to break through the triple challenges of data barriers, algorithm bottlenecks and ethical compliance (23).

Future research should focus on three major directions:

1. Technology integration and innovation: Promote the vertical application of multimodal large models in the field of public health, integrate medical imaging, genomics and social media text, and build a panoramic epidemic intelligence map. At the same time, it strengthens the deployment of edge intelligence and improves the localisation analysis capabilities of resource-poor areas.
2. Interdisciplinary collaboration mechanism: Establish a triangular collaboration platform of “public health-computer science-linguistics” to bridge the gap between technology research and development and prevention and control practice. The successful experience of the “Artificial Intelligence Pharmacy Interdisciplinary Research Center” of the Third Xiangya Hospital in China shows that such collaborations can significantly accelerate technology transformation.
3. Ethics and privacy protection framework: Design a de-identified text processing solution that complies with GDPR, HIPAA, and China’s Personal Information Protection Law, and develop a federated learning architecture to support inter-agency data collaboration without sharing raw data.

At present, public health intelligence systems based on text analysis and NLP are in a critical stage of transformation from “technical validation” to “large-scale application.” As governments incorporate AI public health into their national strategies (such as China’s Guidelines for the Construction of High-level Public Health Colleges), this field is expected to play a more central defensive role in the next round of global public health crises.

The core goal of this study is to build an intelligence analysis system for public health emergencies to achieve:

Multi-source intelligence fusion: Integrate structured case data with unstructured flow control texts.
Risk intelligence identification: Predict the spread path of the epidemic through machine learning models.
Decision support visualization: Generate knowledge graphs and communication chain reports.

Innovatively apply the information life cycle theory of library and information science to epidemic data management, and design a closed-loop framework of “collection→ analysis→ service”.

## 3 Research methodology

Adopt a mixed study design:

1. Quantitative analysis: Chi-square test screens key transmission sites, and logistic regression identifies high-risk groups.
2. Text mining: The LDA topic model extracts the implicit topics of the flow survey report, and the BiLSTM-CRF model extracts the entity relationship.
3. Knowledge graph: TransH algorithm constructs a spatio-temporal propagation network.
4. System development: Build a B/S architecture analysis platform based on the PythonFlask framework.

### 3.1 Literature review

#### 3.1.1 Theoretical application of library and information science in public health events

##### 3.1.1.1 Emergency information management theory

Fu Ping proposed a “four-dimensional model” of library emergency services, including four dimensions: information integration, space services, technical support and collaborative networks. The American Library Association (ALA) practiced the model in COVID-19 through a three-step response mechanism: opening the emergency digital resource library in phase 1; the second stage is to establish a virtual consultation desk; The third stage provides mental health information services. The NHS library in the UK adopts the “intelligence ladder” model, which transforms raw data into seven levels of decision-making knowledge: data→information→ knowledge→ intelligence→ insights→ decision-making → action.

##### 3.1.1.2 User information behavior theory

Zhang Jing’s research found that the user information needs in public health emergencies show a “pyramid structure”: the grassroots public needs protection guidelines (62.3%), medical staff need diagnosis and treatment plans (24.1%), and decision-makers pay attention to the prediction of communication trends (13.6%). The National Library of Singapore has established the Twitter Public Opinion Sentiment Analysis Model (SnowNLP-LDA) to achieve hierarchical response to needs, with an accuracy rate of 78.5%.

#### 3.1.2 Progress in text analysis and NLP technology

##### 3.1.2.1 Feature selection method

The chi-square test (*χ*^2^) in short text classification has become the preferred feature selection method due to its computational efficiency. In the sentiment classification task, chi-square statistics evaluates the correlation between words and categories, and screens out high-discriminant features such as “aggregation” (*χ*^2^ = 15.2) and “hidden” (*χ*^2^ = 12.8). TF-IDF combined with N-Gram can capture key phrases such as “village clinic visit” (2-gram), improving the classification accuracy by 7.3%.

##### 3.1.2.2 Knowledge graph construction

Knowledge graph reasoning algorithms are mainly divided into two categories:

Distance-based translation models: such as TransE, TransH, TransR. Among them, TransH solves the 1-N relationship problem through the projection plane, and the F1 value reaches 0.86 in the relationship prediction of “case→ place” (TransH: embedding dimension = 256, learning rate = 0.01, negative sampling rate = 5, margin parameter *γ* = 1.0, training epochs = 500; LDA: *α* = 0.1, *β* = 0.01, number of topics = 5 (determined by coherence score); BiLSTM-CRF: learning rate = 1e-3, dropout = 0.5, batch size = 32).

Graph propagation-based model: GCN realizes relationship completion through neighborhood node aggregation, and the accuracy is improved by 19% in COVID-19 propagation chain reconstruction (Table 4).

**Table 4:** Comparison of public health emergency intelligence systems.

System name	Core technology	Advantage	Confined
NLMDIMRC	MeSH thescaries	Authoritative Medical Terminology Mapping	No social media integration
United KingdomNHSKFH	Knowledge ladder model	Accurate decision support	Rely on manual annotation
OpenSPG	TransH+GCN	Industrial-grade knowledge reasoning	Not suitable for epidemic scenarios
This study system	Multimodal integration	End-to-end automation	Hardware acceleration required

The existing system mostly focuses on a single data type and lacks in-depth mining of flow control text. Xu Chunhua used decision trees to grade measles events, but did not achieve real-time prediction. This study will fill this gap.

### 3.2 Data sources and processing

#### 3.2.1 Sample set construction

Taking the COVID-19 epidemic in Shijiazhuang City from January to February 2021 as the research object, a total of 1,033 official files of confirmed cases were collected, including:

Structured data: demographic characteristics (gender, age, occupation), clinical information (date of onset, date of diagnosis).

Unstructured text: flow survey report (1,006 copies), emergency response plan (20 copies).

Data preprocessing process:

1. Entity standardization: unify geographical names such as “Xiaoguozhuang Village, Zengcun Town→XGZVillage” and so on.
2. Time parsing: Convert “3 days before onset” to absolute date.
3. Missing value processing: KNN algorithm is used to complete 14.2% of the missing activity trajectory.

#### 3.2.2 Characteristic engineering

Three types of features are constructed based on the literature:

Spatio-temporal characteristics: risk level of residence (high/medium/low), recent gathering activity (yes/no).
Behavioral characteristics: delayed visit (≥3 days), history of contact with primary care institutions (village clinic/individual clinic).
Text features: TF-IDF weighted keywords (e.g., “wedding banquet,” “church”; Table 5).

**Table 5:** Distribution of basic characteristics of samples (*n* = 1,033).

Features	Category	Frequency	Percentage
Gender	Man	339	32.8%
	Female	509	49.3%
Age	<60 years old	286	27.7%
	≥60 years old	747	72.3%
Gathering history	Wedding banquet participation	425	41.1%
	Church Events	218	21.1%
First diagnosis institution	Village Clinic	341	33.0%
	County hospitals	492	47.6%

### 3.3 Analysis method system

#### 3.3.1 Statistical analysis methods

Chi-square test: The chi-square test excels in analyzing “associations between categorical variables”—both transmission venues (e.g., churches, wedding banquets, village clinics) and case infection status are categorical data, formula:


$$x^{2}=\sum_{i=1}^{n}\frac{{\left(0_{i}-E_{i}\right)}^{2}}{E_{i}}$$

Logistic regression: One of the study’s goals is to identify high-risk populations. Logistic regression is suitable for “analyzing influencing factors of binary dependent variables (infected/uninfected).” predict individual infection risk, dependent variable Y = confirmed outcome (1/0), independent variable X = [age, aggregation history,…],


$$\text{logit}(P)=\ln(\frac{p}{1-p})=\beta_{1}+\beta_{1}X_{1}+\beta_{2}X_{2}+\cdots +\beta_{k}\;\;X_{k}$$

#### 3.3.2 Data mining model

*C5.0 decision tree*: The study needs to generate rules for high-risk area identification. The C5.0 decision tree offers advantages of “high interpretability and direct output of classification rules.” Generate high-risk area judgment rules, information gain splitting criteria:

$\text{Gain}(S,A)=\text{Entropy}(S)-\sum_{v\in \text{Values}(A)}\frac{|S_{v}|}{|S|}\text{Entropy}(S_{v})$

*Apriori correlation rule*: The study aims to mine epidemic transmission chain patterns (e.g., associations between “venues, populations, and transmission paths”). The Apriori algorithm is a classic method for association rule mining, which can identify high-frequency co-occurring event combinations (e.g., “church attendance → family gatherings,” “wedding banquet participation → village transmission”) from a large volume of case data. Mine the propagation chain pattern, set the minimum support level of 0.3 and the confidence level of 0.7.

#### 3.3.3 NLP technology stack

*Knowledge graph*: The study needs to construct a spatiotemporal transmission network of “cases-venues-time.” The TransH algorithm addresses the “1-N” relationship problem (e.g., one “church” venue associated with multiple “cases”) that the traditional TransE algorithm struggles with. TransH algorithm models entity relationships, scoring functions:


$$f(h,r,t)=\|h_{⊥}+dr-t_{⊥}\|{{}^{2}}_{2}$$

Thereinto, $h_{⊥}=h-w_{r}^{Τ}{\mathrm{hw}}_{r},t_{⊥}$ Similarly,

*LDA topic model*: The study needs to extract latent topics (e.g., “types of gathering activities,” “treatment-seeking paths”) from 1,006 unstructured epidemiological survey reports. LDA is a classic unsupervised topic modeling method suitable for “mining latent topics from large-scale text.” A small *α* value (0.1) ensures each survey report focuses on a few core topics (avoiding topic dispersion), and a small *β* value (0.01) ensures each topic consists of a few high-frequency keywords (e.g., “wedding banquet,” “church,” “village clinic”). This facilitates clear identification of transmission-related core topics in the text, providing thematic guidance for subsequent entity relationship extraction. Extract the implicit topics of the flow survey report, Dirichlet’s prior parameters *α* = 0.1, *β* = 0.01.

### 3.4 System development and testing

#### 3.4.1 Technical architecture

The system adopts a hierarchical architecture:

Data layer: MySQL stores structured data, and MongoDB stores text.
Analysis layer: PyTorch implements the TransH graph, and Scikit-learn runs the decision tree.
Application layer: The system was implemented using a Python Flask back-end with REST API services and Vue.js for interactive visualization.

#### 3.4.2 Test scheme

Functional testing: Verify the output accuracy of modules such as chi-square test and graph construction.
Performance testing: Simulate response time under thousands of data volumes.
User testing: 10 CDC experts were invited to assess intelligence availability.

### 3.5 System development and implementation

#### 3.5.1 Intelligence collection module

In response to the integration challenges of multi-source heterogeneous data, an intelligent acquisition module is developed:

*Structured data*: Connect to the Hebei Provincial Epidemic Direct Reporting System through API to automatically extract case attribute fields.

*Flow adjustment text*: The rule engine + BiLSTM model is used to extract entity relationships, and the process is as follows:

1. Sentence segmentation: “The patient attended the wedding banquet on January 3 and went to the village clinic on January 5” → [Fragment 1, Fragment 2].
2. Physical identification: wedding banquet →EVENT, village clinic → LOC.
3. Relationship classification: < patients, attendance, wedding banquet >, < patients, medical visits, village clinics, >.

F1-score reached 0.83 on the test set, and the recall rate increased by 29%.

#### 3.5.2 Core analysis module

##### 3.5.2.1 Epidemic risk prediction

Individual infection risk scorecard was constructed based on logistic regression:

Input characteristics: age ≥60 years old (3 points), contact with village clinics (4 points), participation in gathering activities (2 points).
Risk classification: low risk (0–3 points), medium risk (4–6 points), high risk (≥7 points).

The model AUC = 0.79, and the calibration curve showed that the predicted probability matched the actual incidence well (Brier score 0.11).

##### 3.5.2.2 Knowledge graph construction

The TransH algorithm was used to construct the “case-site-time” map:

Entity type: 7 Classes (Patient, Place, Institution, Event, etc.)
Relationship types: 4 categories (located, engaged, contacted, belonging).
Graph statistics: 1,532 nodes and 2,876 edges.

Embedding dimension 256, training loss function:


$$L=\sum_{(h,r,t)\in \tau }\max(0,\;f(h,r,t)+\gamma -f(h\prime ,r,t\prime ))$$

The link prediction accuracy is 89.3%, which is better than TransE (82.1%).

##### 3.5.2.3 Decision tree hierarchy

The C5.0 algorithm is used to generate a high-risk area judgment tree (All classification thresholds were automatically determined by the C5.0 algorithm on the training set through maximizing information gain and their stability was confirmed by 5-fold cross-validation):

Root node: Proportion of village clinic visits within 14 days (≥33.5%).
Secondary node: Frequency of wedding banquets/church activities (≥2 times/week).
Leaf nodes: high risk (red), medium risk (yellow), low risk (green).
The accuracy of the test set is 85.7%, and the key rule is that “the proportion of medical visits is >33.5% and the frequency of activity is >2 → high risk”.

##### 3.5.2.4 Visualization output module

###### 3.5.2.4.1 Spatiotemporal map of propagation chains

Node layout: The Force-directed algorithm optimizes spatial distribution.
Propagation animation: dynamically display the diffusion path according to the onset timeline.
Thermal overlay: Leaflet map rendering area risk level.

###### 3.5.2.4.2 Associate rule matrix

Show the top 5 strong association rules:

1. {Church Service} → {Family Gathering} (Support 0.52, Confidence 0.86).
2. {Wedding banquet} → {Cross-village communication} (support 0.48, confidence 0.82).
3. {Village Clinic Visit} → {Older adults infection} (Support 0.41, Confidence 0.79).

## 4 Results

### 4.1 Identification of key transmission factors

#### 4.1.1 Chi-square test

The test showed that churches and wedding banquet venues were significantly correlated with cluster transmission (*p* < 0.001), which were the core places of super transmission events (Table 6).

**Table 6:** Chi-square test results identifying associations between site type and cluster transmission of COVID-19 in Shijiazhuang (*n* = 1,033).

Type of premises	Incidence of aggregation	*χ*^2^ value	*p*-value
Church	82.67%	45.32	<0.001
Wedding banquet venue	76.84%	38.17	<0.001
Village clinic	63.52%	29.05	<0.001
School	51.20%	12.86	0.003

#### 4.1.2 Logistic regression analysis

The model identified church activities as the strongest associated risk factor (OR = 6.51), followed by a history of contact in village clinics (OR = 4.72) (Table 7).

**Table 7:** Logistic regression results identifying key risk factors for COVID-19 infection in Shijiazhuang.

Variable	*β*	OR(95% CI)	*p*
Age ≥ 60 years	1.147	3.15(2.28–4.36)	<0.001
Village clinic contact	1.552	4.72(3.41–6.54)	<0.001
Wedding banquet participation	1.032	2.81(1.97–4.00)	0.002
Church Events	1.874	6.51(4.22–10.05)	<0.001
Constant	−3.278	0.038	<0.001

### 4.2 Text mining discovery

#### 4.2.1 LDA theme evolution

The distribution of topics in the flow survey report shows:

Early stage of the epidemic (1–3 days): Theme 0 “Wedding Banquet Communication” (weight 32.1%), Theme 1 “Cross-village mobility” (28.7%).
Peak period of the epidemic (4–10 days): Theme 2 “Family gathering” (41.5%), Theme 3 “Medical run” (22.8%).
Epidemic recession period (after 11 days): Theme 4 “Isolation effect” (38.2%).

Reflect the dynamic changes in communication patterns.

#### 4.2.2 Associate rule networks

The strong rules of Apriori algorithm mining reveal:

Core hub: church node degree centrality 0.62, intermediate centrality 0.58.
Critical path: “Church services→ family gatherings→ school transmission”.

Blocking opportunities: Isolated village clinics can reduce the risk of spread by 34.7%.

### 4.3 System test and evaluation

#### 4.3.1 Performance indicators

Intelligence extraction speed: average response time 2.3 s (thousand-level data).
Model accuracy: 89.3% for knowledge graph link prediction and 85.7% for decision tree classification.
Resource consumption: Peak memory usage is 1.2 GB, CPU utilization is 68%.

#### 4.3.2 User evaluation

CDC experts scored (1–5 points) from three dimensions:

Intelligence correlation: 4.32 ± 0.45.
Interface Ease of Use: 3.87 ± 0.62.
Decision support: 4.56 ± 0.38.

The feedback shows that the visualization of the propagation chain significantly improves the efficiency of flow control.

## 5 Discussion

### 5.1 The application value of library and information science theory

This study verifies the applicability of information life cycle theory in emergency information management:

Acquisition stage: BiLSTM entity extraction is used to solve the unstructured problem of flow modulation text.
Analysis stage: Chi-square test and logistic regression are fused to achieve the transition of “description → prediction”.
Service stage: Knowledge graph visualization meets the spatial cognitive needs of decision-makers.

The system design echoes Fu Ping’s “four-dimensional model”: On one hand, it connects to the Hebei Province epidemic reporting system via API to automatically extract structured case data; on the other hand, it employs a pre-set BiLSTM model to process unstructured manual epidemiological investigation reports, completing entity recognition and association classification.

Secondly, the system designs the “Propagation Chain Spatiotemporal Map” in the visualization output module, utilizing Leaflet map to render the regional risk levels (heat overlay), and optimizes the spatial distribution of nodes through force-directed algorithms, dynamically displaying the transmission paths according to the timeline of incidence.

Thirdly, in the text processing phase, the LDA topic model is used to extract implicit themes from the epidemiological investigation reports, while the BiLSTM-CRF model is employed to extract entity associations. In the analysis and modeling phase, the TransH algorithm is utilized to construct a knowledge graph of “case - location - time,” the C5.0 decision tree generates judgment rules for identifying high-risk areas, and logistic regression is used to develop individual infection risk scorecards. Through the collaborative application of multiple techniques, we achieve a technical support role for intelligence analysis in the dimension of “technical assurance”.

### 5.2 Comparative advantages with existing systems

Compared with representative systems such as NLM DIMRC and the Program for Monitoring Emerging Diseases (ProMED), the system developed in this study demonstrates three notable advantages:

(1)  Intelligence granularity: While ProMED primarily offers early outbreak alerts at the regional or institutional level, our system achieves individual-level risk tracing by integrating structured case data and unstructured mobility texts;
(2)  Response timeliness: The analysis of epidemiological survey reports is reduced from 24 h (as in typical manual processes) to 2.5 h;
(3)  Predictive function: The addition of a high-risk area early warning module (C5.0 decision tree) enables proactive intervention.

Furthermore, when compared with industrial knowledge graph systems such as OpenSPG (developed by Alibaba), our system exhibits stronger applicability in epidemic scenarios. Although OpenSPG excels in large-scale generic knowledge reasoning and supports efficient subgraph querying (QPS > 10,000), its performance in modeling “one-to-many” (1-N) relationships—such as one spreading venue (e.g., a church) linked to multiple cases—is limited. In contrast, the TransH algorithm adopted in this study explicitly addresses the 1-N relationship problem by introducing hyperplanes for relation-specific projection, thereby avoiding representation conflicts caused by multiple associations. Empirical results demonstrate that TransH achieves a link prediction accuracy of 89.3% (F1 = 0.89), outperforming graph convolutional network (GCN) models (F1 = 0.85) on the same dataset. This advantage is particularly critical for reconstructing transmission chains where super-spreading venues are connected to numerous cases.

Nevertheless, OpenSPG’s industrial-level knowledge inference capabilities—such as support for complex relational path mining and efficient distributed reasoning—remain advantageous in large-scale knowledge fusion. Future work will integrate such advanced inference engines to enhance the system’s scalability.

### 5.3 Public health decision support suggestions

Based on the findings, a hierarchical response strategy is proposed:

Red high-risk areas (such as Zeng villages and towns): Immediately block churches and wedding banquet places, and transform village clinics into fever sentinel points.
Yellow medium-risk area: limit the scale of gathering (< 50 people), and carry out nucleic acid testing for all staff in primary medical institutions.
Green and low-risk areas: Strengthen the symptom monitoring system and promote online health consultation.

This strategy optimizes resource allocation and avoids economic losses caused by “one-size-fits-all” lockdowns.

### 5.4 Limitations and future directions

#### 5.4.1 Current limitations

Not integrating social media data (e.g., Weibo help messages). The official data relied upon by the current system has inherent coverage gaps, whereas Weibo data can accurately fill these gaps. On one hand, it can compensate for the coverage limitations of official data that is ‘diagnosis-oriented’ by including key groups that have not been recorded; on the other hand, it can supplement the missing ‘pre-diagnosis signals’ in official data, correcting risk assessment biases and making the sample set more comprehensively reflect the true situation of populations related to the epidemic.

Rural dialects affect the accuracy of text parsing. The natural language processing model used in this study is pre-trained on a standard Chinese corpus and lacks annotated data for local dialects. This is likely to result in two types of errors: on one hand, misclassification of entities; on the other hand, incorrect judgment of relationships. Such bias not only reduces the accuracy of individual risk scores but also obscures the real transmission pathways in rural areas, weakening the system’s guiding value for grassroots prevention and control.

The hardware requirements for real-time prediction are high. Firstly, the complexity of the core analysis model is high and requires substantial computing power. Secondly, there is a significant gap between the grassroots hardware configuration and the system requirements, highlighting the high demands.

#### 5.4.2 Future direction

Expand multimodal intelligence sources: access 120 emergency records and pharmacy drug purchase data.
Developed a lightweight version: a mobile-based flow adjustment assistant APP.
Integrate diacal NLP model: improve the entity recognition effect of Hebei dialect area.

The “Information Lifecycle Theory” of library and information science guides the design of the entire process from data collection to knowledge services. NLP technology is a key tool for information extraction and semantic understanding, and together they support the structuring and visualization of intelligence on a cognitive level.

This system provides a theoretical paradigm and technical path for library and information science to empower public health emergency management, which is of positive significance for improving the national public health emergency system.

## 6 Conclusion

This study successfully developed and validated a text-based and NLP-based public health emergency intelligence system, with major contributions including:

Constructing an interdisciplinary methodological framework: integrating library and information science, epidemiology and artificial intelligence technology.
Reveal the transmission mechanism of the epidemic in Shijiazhuang: churches and wedding banquets are the key diffusion nodes, and the village clinic is the amplifier.
Develop deployable software tools: The system response speed meets the needs of front-line flow adjustment.
The empirical evidence shows that the system significantly improves the efficiency of intelligence sequencing (47.8%), and the accuracy of identification of high-risk areas (85.7%) reaches the leading level in China.

## Data Availability

The datasets presented in this study can be found in online repositories. The names of the repository/repositories and accession number(s) can be found in the article/supplementary material.
